# Fibroblast-Derived MMP-14 Regulates Collagen Homeostasis in Adult Skin

**DOI:** 10.1016/j.jid.2016.03.036

**Published:** 2016-08

**Authors:** Paola Zigrino, Jürgen Brinckmann, Anja Niehoff, Yinhui Lu, Nives Giebeler, Beate Eckes, Karl E. Kadler, Cornelia Mauch

**Affiliations:** 1 Department of Dermatology and Venerology, University of Cologne, Cologne, Germany; 2 Department of Dermatology, University of Lübeck, Lübeck, Germany; 3 Institute of Virology and Cell Biology, University of Lübeck, Lübeck, Germany; 4 Institute of Biomechanics and Orthopaedics, German Sport University and Cologne Center for Musculoskeletal Biomechanics, University of Cologne, Cologne, Germany; 5 Wellcome Trust Centre for Cell-Matrix Research, Faculty of Life Sciences, University of Manchester, Manchester, UK

**Keywords:** ECM, extracellular matrix, MMP, matrix metalloproteinase, Sf, stromal fibroblast, T, time, TGF, transforming growth factor

## Abstract

Proteolytic activities in the extracellular matrix by the matrix metalloproteinase (MMP)-14 have been implicated in the remodeling of collagenous proteins during development. To analyze the function of fibroblast-derived MMP-14 in adult skin homeostasis, we generated mice with inducible deletion of MMP-14 in the dermal fibroblast (MMP-14^Sf–/–^). These mice are smaller and display a fibrosis-like phenotype in the skin. The skin of these mice showed increased stiffness and tensile strength but no altered collagen cross-links. In vivo, we measured a significantly increased amount of collagen type I accumulated in the skin of MMP-14^Sf–/–^ mice without an increase in collagen fibril diameters. However, bleomycin-induced fibrosis in skin proceeded in a comparable manner in MMP-14^Sf+/+^ and MMP-14^Sf–/–^ mice, but resolution over time was impaired in MMP-14^Sf–/–^ mice. Increased accumulation of collagen type I was detected in MMP-14^Sf–/–^ fibroblasts in culture without significant enhancement of collagen de novo synthesis. This points to a degradative but not synthetic phenotype. In support of this, MMP-14^Sf–/–^ fibroblasts lost their ability to process fibrillar collagen type I and to activate proMMP-2. Taken together, these data indicate that MMP-14 expression in fibroblasts plays a crucial role in collagen remodeling in adult skin and largely contributes to dermal homeostasis underlying its pathogenic role in fibrotic skin disease.

## Introduction

Matrix metalloproteinases (MMPs) play a crucial role in the maintenance of the normal balance between extracellular matrix (ECM) synthesis and degradation in tissues. This class of proteolytic enzymes are Zn-dependent endopeptidases, subdivided according to their structural characteristics in secreted and membrane-bound MMPs (MT-MMPs). They display a certain degree of substrate specificity but can often compensate for each other for the processing of some substrates (Nagase et al., 2006). This became particularly evident with the generation of mice models of MMP deficiency. However, unlike most MMP-deletion mice, MMP-14 ablation leads to a severe phenotype, indicating that some activities of this protease in vivo cannot be replaced by additional members of this family. Mice deficient for MMP-14 suffered from osteopenia, ankylosis, and progressive fibrosis of soft tissues (Holmbeck et al., 1999, Zhou et al., 2000). Recently, a model of MMP-14 deficiency has been generated; similar to the previous model, mice die early because of cardiac defects, and only a few older mice could also display some collagen type I accumulation in the skin (Gutierrez-Fernandez et al., 2015). Thus, despite the fact that degradation of interstitial collagen fibrils could be catalyzed, mostly in vitro, by several MMPs (Fields, 2013), MMP-14 (also known as MT1-MMP) seems to be the pivotal collagenase in vivo. In skin, collagens I and III are found in the dermis, with collagen I been the most abundant. At birth, collagen type III is highly expressed in skin, and over time it is reduced, leading to a late increased ratio of type I to III (Gelse et al., 2003). Collagen type I homeostasis in tissues is finely regulated by a continuous balance of synthesis and degradation, the alteration of which leads to various pathologies such as scleroderma, keloid, hypertrophic scar formation, and skin degeneration during aging (Ogawa and Hsu, 2013). Unexpectedly, most experiments with MMP knockout mice show protection from bleomycin-induced fibrosis rather than increased collagen accumulation. The most convincing evidence for increased fibrosis resulting from deletion of an MMP comes from studies with MMP-14–deficient mice (Holmbeck et al., 1999). To further analyze the functional role of MMP-14 in adulthood we generated a conditional knockout of MMP-14 in stromal fibroblasts. To this end, we crossed MMP-14 floxed mice with Coll α2(I) ER-Cre deleter mice. Upon deletion of MMP-14 in adult mice fibroblasts, we could detect a marked skin phenotype with enhanced dermal thickness and tissue stiffness but no alterations in other organs. This phenotype was caused by altered balance between collagen type I synthesis and degradation, with degradation being significantly impaired.

## Results

### Generation of mice with fibroblast-specific depletion of MMP-14 expression

**Figure 1:**
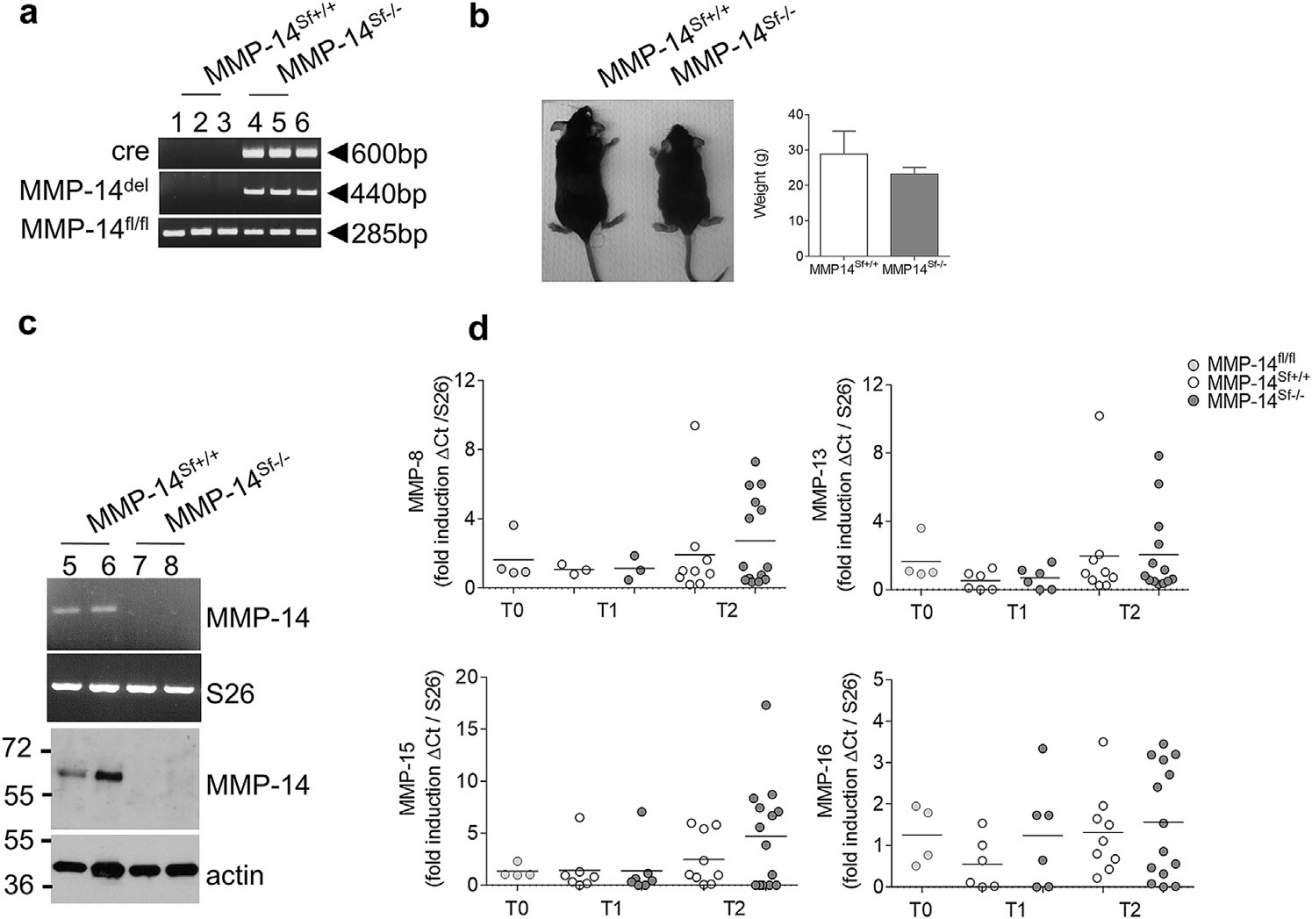
**Fibroblast-specific depletion of MMP-14.** (**a**) Genotyping PCR images used to amplify the LoxP-flanked exons 2–4 of the MMP-14 gene and the PCR product detected only upon LoxP recombination. The lengths (in base pairs) of the amplified PCR fragments are shown. (**b**) Mice after tamoxifen-induced MMP-14 deletion at age 13 weeks (T2). Average weight of sex- and age-matched mice is shown (n ≥ 7). (**c**) MMP-14 transcripts amplification in fibroblasts isolated from adult mouse skin (5 weeks after tamoxifen). Immunoblot analysis of MMP-14 expression in isolated primary fibroblasts. Actin was used as a control. (**d**) Real-time PCR amplifications from skin specimens collected at T0, T1, and T2. Each dot represents one specimen/mouse. S26 was used as control. bp, base pairs; cre, Cre transgene; Ct, cycle threshold; del, deletion; fl, floxed; MMP, matrix metalloproteinase; Sf, stromal fibroblast; T, time.

To explore the functional significance of fibroblast MMP-14 in skin homeostasis, we generated a mouse strain with an inducible fibroblast-specific depletion of MMP-14, which we refer to here as MMP-14 stromal fibroblasts –/– (MMP-14^Sf–/–^). For this purpose, mice carrying the LoxP-flanked exons 2–4 of the MMP-14 gene (MMP-14^Sf+/+^]) were mated with mice carrying the Cre-ERT fusion transgene under the control of a minimal collagen-α2 type I promoter and enhancer driving fibroblast-specific and inducible Cre expression (Sonnylal et al., 2007, Zheng et al., 2002). At 4 weeks of age, mice were fed for 4–5 weeks with tamoxifen. We confirmed in vivo deletion by analysis of genomic DNA, detecting the floxed allele (fl/fl; MMP-14^Sf+/+^), the presence of the Cre gene (*cre*) and a fragment of the MMP-14 gene detectable upon deletion of exons 2–4 (del; MMP-14^Sf–/–^) (Figure 1a). MMP-14 deletion was specifically detected in dermis but not in epidermis where keratinocytes, melanocytes, Langerhans cells, and subsets of T cells are present (see Supplementary Figure S1a online). Upon MMP-14 deletion in fibroblasts, mice at 13 weeks of age developed smaller than floxed littermates, with enlarged extremities and tail, but comparable weight (Figure 1b). The skin was tight, thickened, and less elastic.

In dermal fibroblasts isolated from skin of MMP-14^Sf–/–^ mice we could not detect MMP-14 transcripts or protein (Figure 1c). On the contrary, MMP-14 expression was retained in isolated skin keratinocytes of both mouse genotypes (see Supplementary Figure S1b). Moreover, real-time PCR analysis of RNA isolated from MMP-14^Sf–/–^ and MMP-14^Sf+/+^ skin failed to show significant changes in the collagenases MMP-8 and -13 and in MMP-15 and -16 (*P* = 0.2 at time [T] 1; *P* = 0.9 at T2) (Figure 1d).

### Increased dermal thickness and fibrillar collagen accumulation in MMP-14^Sf–/–^ mice

**Figure 2:**
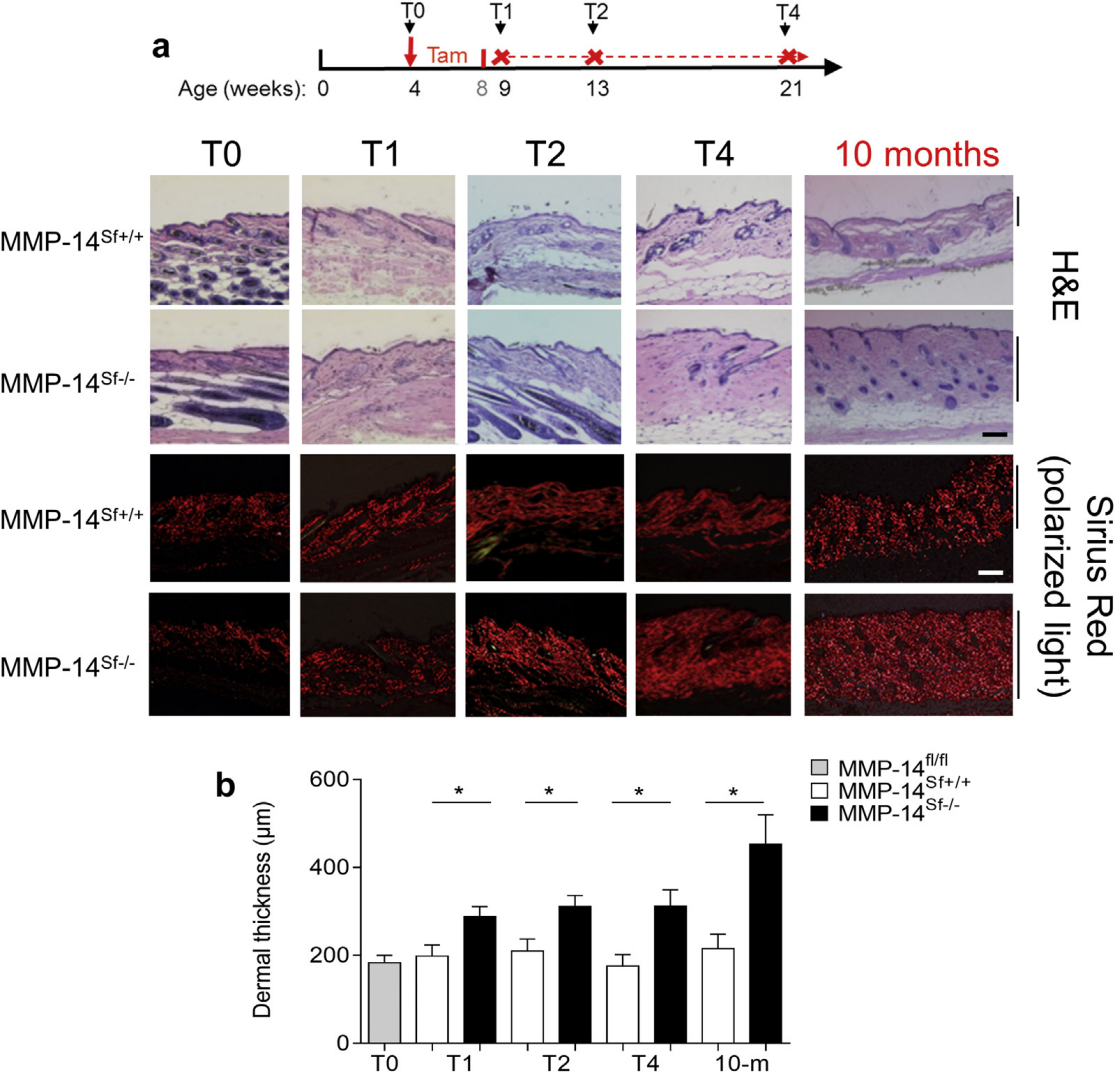
**Increased dermal thickness over time in MMP-14^Sf–/–^ mice.** (**a**) Hematoxylin and eosin and Sirius red staining (analyzed with polarized light) of back skin from mice at different time points after tamoxifen activation of Cre recombinase. The scheme shows the protocol used for tamoxifen induction and the analyzed time points post-tamoxifen, which for simplicity were named as T0 = before tamoxifen diet; and T1 = 1 week, T2 = 5 weeks, and T4 = 13 weeks after feeding protocol was completed; and at the age of 10 months (8 months post-tamoxifen). Scale bar = 100 μm. (**b**) At each time point, skin sections (6 μm) were cut and stained with hematoxylin and eosin, and thickness of dermis was measured microscopically and expressed as average thickness ± standard deviation (^∗^*P* ≤ 0.01, n = 4–13). fl, floxed; H&E, hematoxylin and eosin; m, months; MMP, matrix metalloproteinase; Sf, stromal fibroblast; T, time.

In the skin of mice before tamoxifen feeding (T0), skin morphologies of MMP-14^Sf+/+^ and MMP-14^Sf–/–^ mice were comparable (Figure 2a). After supplying tamoxifen, mice developed progressive dermal thickening within 1 week after Cre activation protocol was terminated (T1), with a significant increase in skin thickness (*P* = 0.01) compared with control littermates (Figure 2a and b). Skin thickening further progressed after 5 (T2, *P* = 0.02) and 13 (T4, *P* = 0.02) weeks and was still detected at 10 months of age (Figure 2a and b). A slightly reduced subcutaneous fat layer, but no gross abnormalities in the epidermal structures, were detected. Histochemical analysis using picrosirius red staining and polarized light microscopy (Junqueira et al., 1978) showed increased collagen fibril accumulation and formation of large bundles in the dermal compartment of MMP-14^sf–/–^ mice (Figure 2a). Even though MMP-14 deletion was detected by genotyping of organs, no significant collagen accumulation was detected in the lungs or in the liver, aorta (see Supplementary Figure S1c and d), kidneys, and heart until 10 months of age (not shown). In MMP-14 full-knockout mice, a phenotype resembling early aging has recently been described (Gutierrez-Fernandez et al., 2015, Holmbeck et al., 1999, Zhou et al., 2000), but in skin of older mice (10 months of age), MMP-14^Sf–/–^ expression of p21, a direct target of p53-induced senescence, was not altered (see Supplementary Figure S2b online).

**Figure 3:**
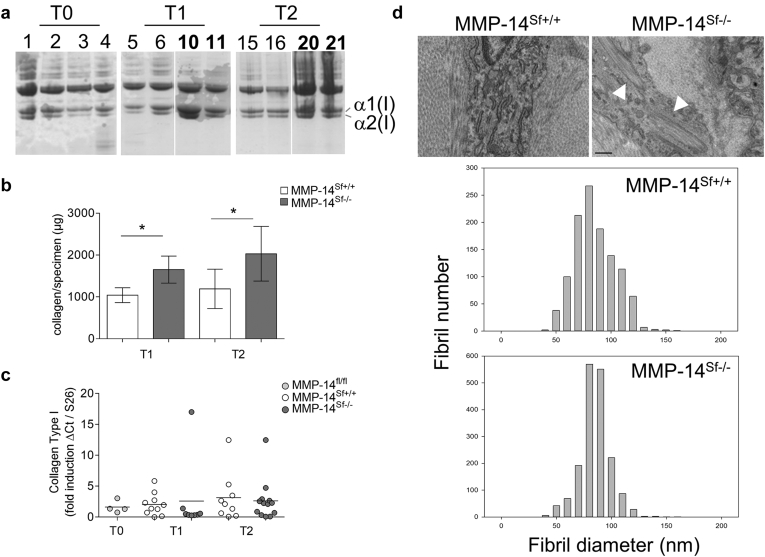
**Accumulation of collagen type I in skin of MMP-14^Sf–/–^ mice.** (**a**) Equal amounts of extracts from the back skin of MMP-14^Sf+/+^ and MMP-14^Sf**–/–**^ mice were resolved by SDS-PAGE and stained with Coomassie blue. Numbers indicate the different mice; boldface (10, 11, 20, and 21) indicates those from MMP-14^Sf**–/–**^ mice. Extracts 3 and 4 are from MMP-14 fl/fl^Cre+^ mice before tamoxifen treatment. (**b**) Collagen content was analyzed in hydrolyzed specimens. Mean values are shown (^∗^*P* ≤ 0.02, n = 4–5). (**c**) Collagen type I transcripts in skin specimens; each dot represents one specimen/mouse. S26 was used for normalization of amplified transcripts. (**d**) Electron microscopy of skin sections at T2 showing a comparable diameter of fibrils in both mice. The number on the axis refers to the number of analyzed fibrils. Scale bar = 0.5 μm. Ct, cycle threshold; fl, floxed; MMP, matrix metalloproteinase; Sf, stromal fibroblast; T, time.

A strong increase in the amount of acetic acid/pepsin-extracted skin collagen was visible by SDS-PAGE (Figure 3a). In addition, hydroxyproline content analyzed in skin biopsy samples from four age- and sex-matched MMP-14^Sf+/+^ and MMP-14^Sf–/–^ mice showed a significant increase at both analyzed time points (T1, *P* = 0.01; T2, *P* = 0.02) (Figure 3b) in MMP-14^Sf–/–^ compared with MMP-14^Sf+/+^. At these time points, the levels of α(I) collagen transcripts were comparable in both mouse genotypes (Figure 3c). Further, real-time analysis of collagen type III, fibronectin, and α-smooth muscle actin, associated with skin fibrosis and induced by the profibrotic transforming growth factor (TGF)-β (Jinnin, 2010, Perlish et al., 1988, Varga et al., 1987), also did not show any significant changes of transcripts at both T1 and T2 (see Supplementary Figure S2a) and in 10-month-old mice (see Supplementary Figure S2b). In agreement with these data, total amounts of TGF-β1 were not altered in skin extracts, and active TGF-β1 was undetectable (see Supplementary Figure S2c). To investigate whether accumulation in collagen type I also results in alterations in the formation of fibrils, electron microscopy studies were performed on the dermis of MMP-14^Sf–/–^ and MMP-14^Sf+/+^ mice. The dermis of the both animals contained collagen fibrils with a relatively restricted and comparable distribution of diameter (average size = 81 ± 17 and 79 ± 12 in MMP-14^Sf+/+^ and MMP-14^Sf–/–^ mice) (Figure 3d). Therefore, the fibrotic phenotype in MMP-14–deficient mice was the result of elevated collagen levels and not of an increase in fibril diameters. In the skin of MMP-14^Sf–/–^ mice we also detected large intracellular bundles of collagen in fibroblast-like cells (Figure 3d, arrowheads).

### MMP-14^Sf–/–^ mice display increased tissue stiffness but no altered collagen cross-linkage, vascular stability, or inflammation

**Figure 4:**
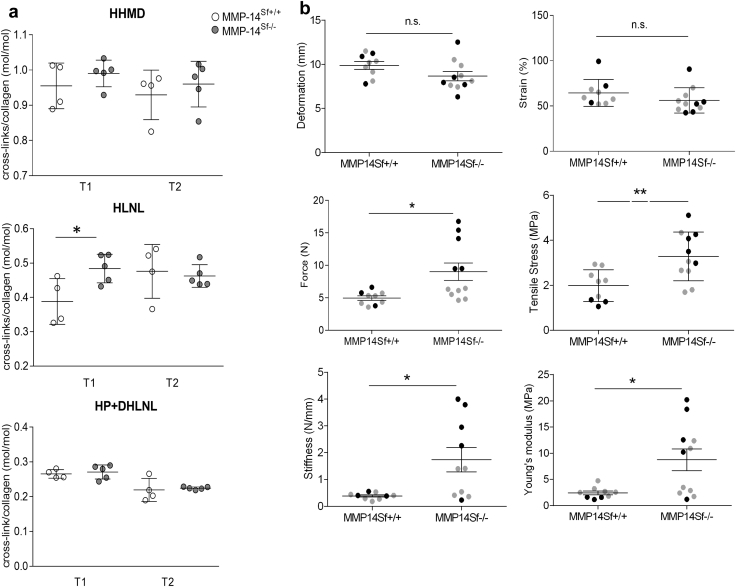
**Altered mechanical properties, but not cross-link types, in skin from MMP-14^Sf–/–^ mice.** (**a**) Analysis of collagen cross-link formation in skin samples; depicted is the average ratio of HP + DHLNL to HLNL crosslinks (n ≥ 4). (**b**) Different mechanical properties were determined in back skin specimens freshly prepared from MMP-14^Sf+/+^ and MMP-14^Sf–/–^ mice at T2 (^∗^*P* ≤ 0.01,^∗∗^*P* ≤ 0.001). Each dot represents one specimen/mouse; black dots = males, grey dots = females (n ≥ 9). HHMD, Lys aldehyde-derived cross-link histidinohydroxymerodesmosine; HLNL, hydroxylysinonorleucine; DHLNL, dihydroxylysinonorleucine; HP, hydroxylysyl pyridinoline; MMP, matrix metalloproteinase; n.s., not significant; Sf, stromal fibroblast.

In systemic sclerosis, the increased deposited collagen shows hydroxylysine aldehyde-derived collagen cross-links that are more typical of cartilage and bone (Brinckmann et al., 2001). To investigate whether the skin phenotype in MMP-14^Sf–/–^ mice resembles this feature of a sclerotic disease, we analyzed cross-links in the skin of age- and sex-matched MMP-14^Sf–/–^ and MMP-14^Sf+/+^ mice. We did not detect any increase in hydroxylysyl pyridinoline and its precursor, dihydroxylysinonorleucine, which is typical for hard tissues (Brinckmann et al., 2001). In addition, there were no changes in the concentration of the Lys aldehyde-derived cross-link histidinohydroxymerodesmosine. The cross-linked hydroxylysinonorleucine showed a significant increase at T1 but not T2 (Figure 4a). Despite the fact that collagen cross-links are not altered, the excessive deposition of collagen and the increased fibril number may alter the mechanical properties of the skin in MMP-14^Sf–/–^ mice. Ultimate force, ultimate stress, and Young’s modulus in MMP-14^Sf–/–^ mouse skin were found to be significantly higher than in MMP-14^Sf+/+^ skin (*P* = 0.01, *P* = 0.006, and *P* = 0.01, respectively). In addition, MMP-14 deletion in skin fibroblasts led to significant stiffening (*P* = 0.01) of the tissue, despite deformability being comparable in both mouse genotypes (Figure 4b).

Tissue damage, initially involving the endothelium and inflammation, are critical initiators of skin fibrosis (Denton et al., 2006). In mice lacking MMP-14 in fibroblasts, we could not identify any vascular damage, either by immunofluorescence assessment and quantification of blood vessels (CD31) or by injection of fluorescent dextran used to assess vascular leakage (see Supplementary Figure S3 online). In addition, we did not find any significant alteration in lymphocytic or myeloid cell infiltration before or at the time of dermal thickening in the tissues indicative of enhanced inflammation (data not shown).

### Increased accumulation of collagen in MMP-14^Sf–/–^ mouse skin in vivo and in fibroblasts in vitro

**Figure 5:**
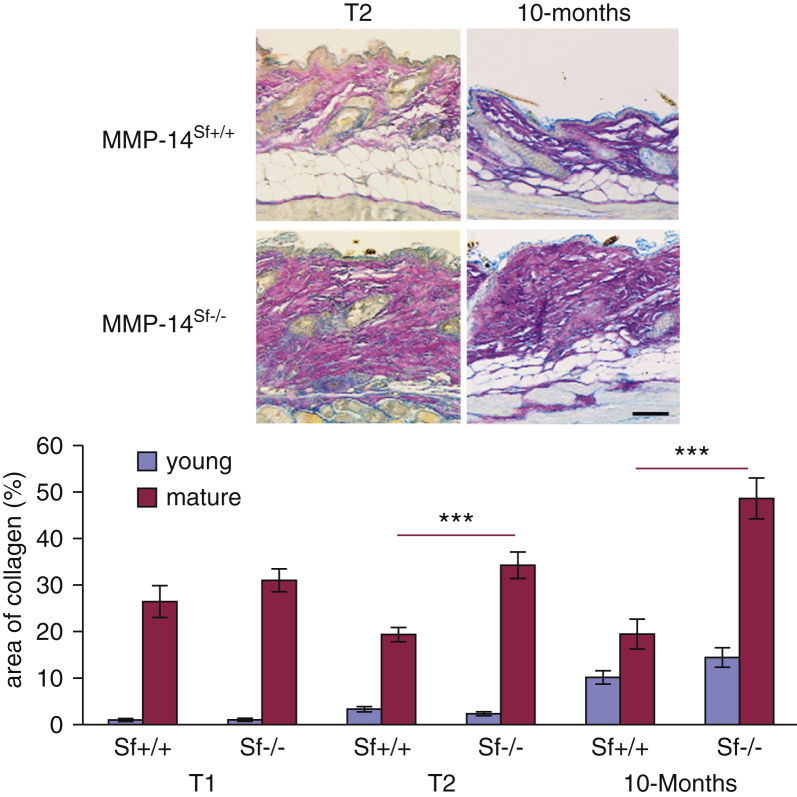
**Mature collagen is significantly accumulated in skin of MMP-14^Sf–/–^ mice.** Herovici’s staining of skin at the two indicated time points was analyzed by light microscopy. Colors on images were inverted and split into single channels to separately assess the amount of blue (young, collagen type III) or pink (mature, collagen type I) collagen fibrils. Quantification of the average of both colors is shown on the right (n = 10 at T1 and T2; n = 6 at 10 months, each for MMP-14^Sf+/+^ and MMP-14^Sf–/–^). ***P < 0.001. MMP, matrix metalloproteinase; Sf, stromal fibroblast; T, time.

**Figure 6:**
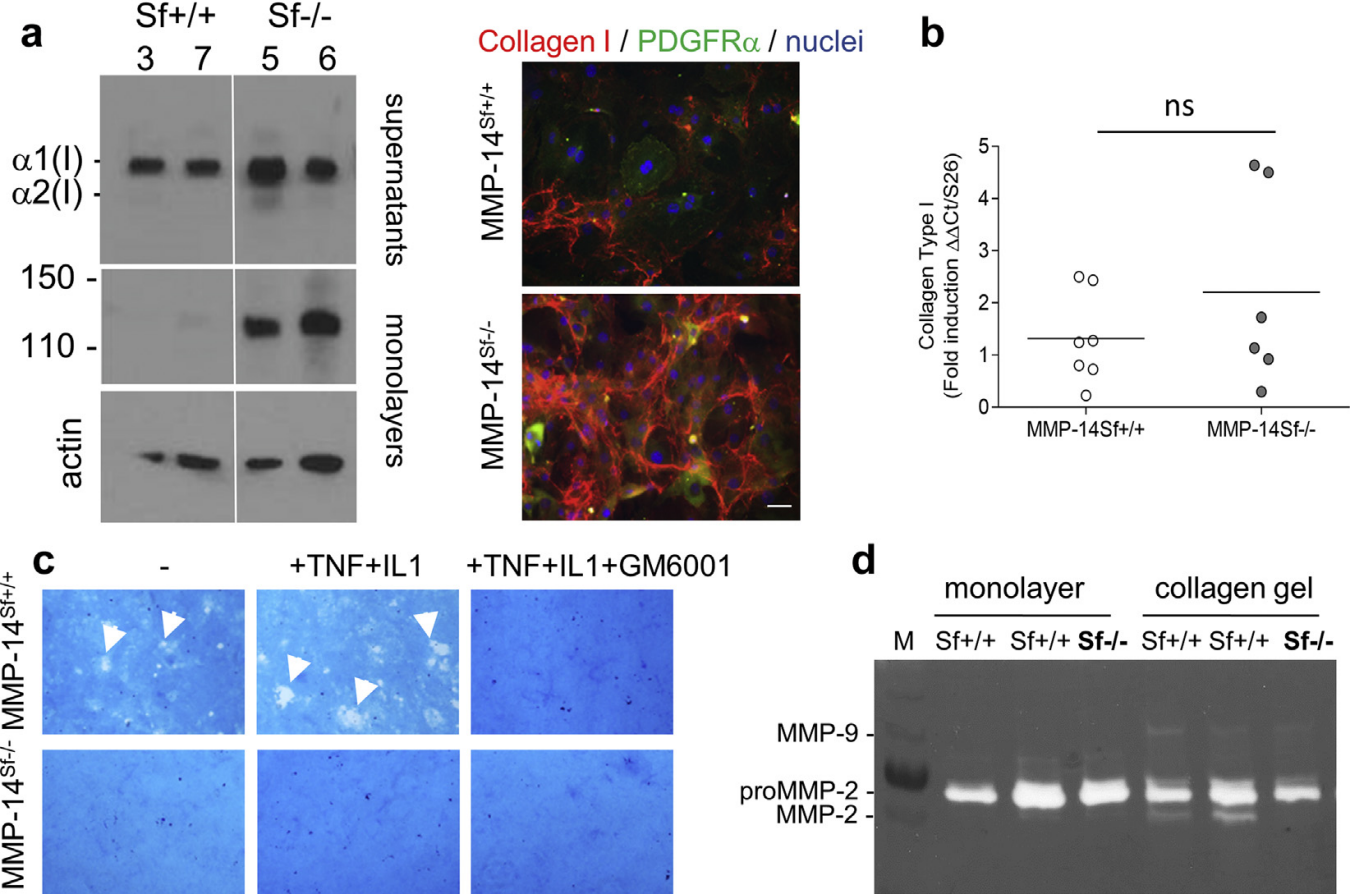
**MMP-14 is the main collagenolytic enzyme in adult skin fibroblasts.** (**a**) Fibroblasts from two different mice per genotype (Sf+/+, mice 3 and 7) and (Sf–/–, mice 5 and 6) were cultured as monolayers for 4 days. Collagen type I was detected in immunoblots (10 μg) and by immunofluorescence (right). Actin was used as control. Scale bar = 100 μm. (**b**) Collagen type I transcripts in independent cultures of fibroblasts. (**c**) Collagenolytic activity of fibroblasts seeded on a reconstituted type I collagen film in the absence (-) or presence (+) of tumor necrosis factor, IL-1, or GM6001 inhibitor. Collagen degradation is detected as white areas on a blue background (arrowheads). The experiment was repeated twice in triplicate. (**d**) Gelatin zymography of supernatants (normalized to the cell lysates) from Sf^+/+^ and Sf^–/–^ cultured as indicated. Ct, cycle threshold; M, marker; MMP, matrix metalloproteinase; ns, not significant; PDGFR, platelet-derived growth factor receptor; Sf, stromal fibroblast; T, time; TNF, tumor necrosis factor.

To visualize and analyze the distribution of dermal collagen we performed classic histochemical stainings based on Herovici’s protocol, which stains collagen dark pink (mature [collagen type I]) and blue (young [collagen type III]) in human and mouse skin (Collins et al., 2011, Fitzgerald et al., 1996, Watt and Fujiwara, 2011). Whereas the amount of young collagen (blue) over time was quite comparable in both mouse genotypes, the amount of mature collagen (dark pink) was significantly increased in the skin of MMP-14^Sf–/–^ compared with control mice (Figure 5). To directly address whether increased collagen results from impaired degradation, we isolated fibroblasts from these mice and analyzed them in culture. MMP-14^Sf–/–^ fibroblasts accumulated collagen type I protein in both supernatants and cell layer. Increased collagen type I was also visible by immunostaining of monolayer cultures (Figure 6a). De novo synthesis of collagen type I, as detected by real-time PCR, was not significantly altered, although transcripts were slightly increased (Figure 6b).

One target of MMP-14 is proMMP-2, which is also involved in downstream processing of collagen type I and whose activation is induced by culturing fibroblasts in three-dimensional collagen lattices (Fields, 2013, Zigrino et al., 2001). When cultured in collagen lattices, fibroblasts from MMP-14^Sf–/–^ mice did not lose their capacity to contract the gel (see Supplementary Figure S4a online) but almost completely lost the ability to activate proMMP-2, whereas this was retained in MMP-14^Sf+/+^ fibroblasts (Figure 6d, and see quantification in Supplementary Figure S4b). Using an in vitro degradation assay (Ruangpanit et al., 2002), we could detect collagen degradation areas (in white) by fibroblasts from MMP-14^Sf+/+^ cultured on a layer of fibrillar collagen. This was further enhanced by the presence of two inducers of MMP expression/activity, tumor necrosis factor and IL-1, and abolished by the MMP inhibitor GM6001 (Figure 6c). However, in normal culture conditions or in the presence of the inducers we could not detect any degradation of fibrillar collagen by MMP-14^Sf–/–^ fibroblasts (Figure 6c and see quantification in Supplementary Figure S4c).

### Comparable collagen synthesis in bleomycin-induced fibrosis and in wound repair

A murine model of skin fibrosis induced by subcutaneous injection of bleomycin has been described (Yamamoto et al., 1999). Based on this model system, skin lesions generated in MMP-14^Sf–/–^ and MMP-14^Sf+/+^ mice showed dense accumulation of collagen in the dermis and replacement of subcutaneous fat by connective tissue (see Supplementary Figure S5 online). In both mouse genotypes, the dermal thickness of bleomycin lesions was increased to the same twofold extent over control saline-injected sites, indicating that collagen production is comparable in MMP-14^Sf–/–^ and MMP-14^Sf+/+^ mice. By Herovici’s staining we detected a significant increase in collagen type I and III upon bleomycin stimulation of both mouse genotypes (see Supplementary Figure S5). Interestingly, when mice were left to recover for 4 weeks after bleomycin treatment, skin thickness and amount of collagen type I were significantly reduced in MMP-14^Sf+/+^ but not in MMP-14^Sf–/–^ mice (see Supplementary Figure S6a and b online).

A temporarily and locally restricted fibrotic reaction is also observed during tissue repair. To investigate the physiological role of fibroblast MMP-14 in wound-healing responses, we generated excisional skin wounds on the backs of 8-week-old MMP-14^Sf–/–^ and MMP-14^Sf+/+^ littermates. Wound closure was followed over time and expressed as a percentage of the initial wound area. The kinetics of wound repair were comparable in both mice (see Supplementary Figure S7a online). Histological examination of wounds at day 11 postwounding indicated a comparable formation of granulation tissue in quantity (see Supplementary Figure S7b) and collagen deposition (Sirius red stain). Collagen type I and III were present in the granulation tissue at day 13 with prevalence of the collagen III (see Supplementary Figure S7c). In addition, invasion of vascular structures, as shown by CD31 staining, was comparable in both mouse systems (see Supplementary Figure S7c).

## Discussion

Metalloproteinases have been implicated in direct or indirect remodeling of the ECM. In vivo analysis of MMP deficiency has shown that the activity of most MMPs can be efficiently compensated (Page-McCaw et al., 2007). However, in case of MMP-14, constitutive deletion leads to early postnatal death within 3 weeks (Gutierrez-Fernandez et al., 2015, Holmbeck et al., 1999, Zhou et al., 2000). Only a few mice that reached older age also presented with a local accumulation of collagen in the skin (Holmbeck et al., 1999). Using mice with conditional knockout of MMP-14 in stromal fibroblasts, we showed that ablation of MMP-14 in fibroblastic cells in adult skin results in a fibrotic skin phenotype. This effect was limited to the skin and was not detected in the kidney, liver, and lungs, although collagen α2 (I) Cre promoter was shown to be active in fibroblasts in the lungs and kidney (Zheng et al., 2002). A possible explanation is that activity of MMP-14 in collagen turnover in these organs is not relevant or is efficiently replaced by other collagenases. In lung, for instance, during embryonal development expression of MMP-14 and -2 is high, but both taper off with the completion of lung development (Greenlee et al., 2007). Additional collagenases may exert a major role in lung homeostasis, possibly MMP-8 and MMP-13, which are also found to be induced in chronic obstructive pulmonary disease (Woode et al., 2015), or MMP-1, which is absent in fibroblast foci from idiopathic pulmonary fibrosis (Pardo and Selman, 2012). In skin, MMP-8 and MMP-13 collagenolytic activity may be of secondary importance to MMP-14, because mice deleted for the single enzymes failed to show any constitutive skin phenotype even in adulthood (Page-McCaw et al., 2007).

Collagen type I turnover in vivo in skin is reported to be quite long, with an estimated half-life of approximately 15 years in human and 74–80 days in rat and mouse skin (Ohuchi and Tsurufuji, 1970, Rucklidge et al., 1992, Verzijl et al., 2000). Collagen accumulation in skin of MMP-14^Sf–/–^ mice was detected up to 10 months of age and was not accompanied by significant alterations in de novo synthesis, indicating that impaired degradation may alter collagen homeostasis and thus collagen half-life. Electromicroscopy analysis of MMP-14^Sf–/–^ mouse skin at different stages of progressive fibrosis did not detect alterations in fibrils or collagen bundle form and size compared with controls. A large accumulation of collagen type I is typically detected in fibrotic tissue, where normal architecture is replaced with a largely acellular, collagen-rich, stiff connective tissue (Bhattacharyya et al., 2012). In agreement, the skin of MMP-14^Sf–/–^ mice had increased tissue tensile strength, stress, and stiffness and Young’s modulus compared with control animals. Even though an increased ratio of posttranslational collagen modifications have also been implicated in altering mechanical properties of the skin, we could not detect any major alteration in the amounts or pattern of collagen cross-links typically observed in human sclerosis and bleomycin-induced skin fibrosis in mice (Brinckmann et al., 2001, Willenborg et al., 2014, Yamamoto et al., 1999).

Mouse models of sclerotic skin diseases comprise inflammation-dependent (bleomycin model) and inflammation-independent types. The latter can be subdivided into models with higher TGF-β levels or a lack of collagen degradation (Denton and Abraham, 2004, Yamamoto et al., 1999). Further, fibrosing skin diseases, such as human scleroderma, are thought to stem from vascular defects and enhanced inflammatory reactions (Denton et al., 2006). In MMP-14^Sf–/–^ mouse skin at stages before and after significant dermal thickening, we did not detect obvious abnormalities in blood vessels and in the amount of inflammatory cells infiltrating the tissue. These data, together with the lack of cross-link alterations, indicate that lack of MMP-14 in adult skin induces a type of fibrosis due to reduced degradation but not to inflammation.

Complete deletion of MMP-14 was previously shown to result in decreased levels of total TGF-β1 signaling and enhanced vascular leakiness (Sounni et al., 2010). On the contrary, in a recently generated model of MMP-14 deficiency, a significant increase in TGF-β1 expression levels was detected (Gutierrez-Fernandez et al., 2015). In the fibroblast-specific MMP-14 knockout model, we could not find alterations in TGF-β1 levels in skin; in agreement, vessel permeability to dextran was comparable to that of wild types, and collagen synthesis also was not altered. This would imply that, unlike fibroblast MMP-14–deletion mice, the effect detected in constitutive MMP-14 knockout mice may result from alterations in cell types other than fibroblasts in tissue.

Additional possible explanations are that, in the absence of MMP-14, processing of additional factors or receptors is altered. These may in turn contribute to counteract alterations in TGF-β signaling. Endoglin, for example, is a transmembrane protein acting as a TGF-β auxiliary receptor. Either as full length or in soluble form (spliced or the MMP-14–shed form), endoglin may affect differential TGF-β1 signaling or CCN2 (alternative name, connective tissue growth factor, or CTGF) synthesis and thus positively or negatively affect subsequent signaling (Maring et al., 2012). Surprisingly, despite the increased skin stiffness we detected in MMP-14^Sf–/–^ mice, the number of α-smooth muscle actin–positive myofibroblasts, whose differentiation can be driven by the mechanical properties of the surrounding matrix (Hinz, 2009), was not altered. However, this correlated with unaltered levels of TGF-β1 and possibly indicates that matrix modifications such as collagen cross-links, which were not altered in our system, are necessary, together with increased collagen, for the induction of myofibroblast differentiation.

However, our data indicate that lack of MMP-14 represents a pathogenic mechanism of fibrotic disease, which is independent of TGF-β, inflammation, and vascular damage. This was supported by the observation that in two models of fibrosis in mice, namely bleomycin induced and wound healing (scar formation), we could not observe any major alterations compared with control mice. In agreement with previous studies showing restoration of dermal thickness after bleomycin treatment as result of proteolysis (van der Slot-Verhoeven et al., 2005), dermal thickening was diminished after a resting period in wild-type mice but not in MMP-14^Sf–/–^ mice. This would corroborate the major role for MMP-14 in collagen degradation in skin.

Lack of alterations in granulation tissue formation during tissue repair may suggest that this process is independent of fibroblast-derived MMP-14. At this late stage, macrophages in granulation tissue may clear excess ECM in the scar (Rohani et al., 2015, Rohani and Parks, 2015). Because we did not detect alterations in scar formation in the macrophage MMP-14 mouse mutants (Klose et al., 2013), it is conceivable that collagenolytic activity in scar derives from another enzyme, possibly MMP-13, that is pivotal in this process in liver fibrosis (Fallowfield et al., 2007).

In line with the observations we made in vivo, increased accumulation of collagen type I was also detected in MMP-14^Sf–/–^ fibroblasts in culture with no significant enhancement of collagen de novo synthesis, pointing once more to a degradative but not synthetic phenotype. In vitro, MMP-14^Sf–/–^ fibroblasts lost their ability to process fibrillar collagen type I and to activate proMMP-2. These observations were made also for MMP-14 knockout mice, in which no alteration in skin in vivo was detected. This was likely because of the early postnatal death of the mice (Holmbeck et al., 1999). Our analysis shows that in adult skin, MMP-14 is the collagenolytic enzyme responsible for collagen remodeling. MMP-14 has a pivotal role in adult collagen homeostasis, and inhibition of its expression and activity may contribute to pathogenesis of fibrosis.

## Materials and Methods

### Generation of fibroblast-specific MMP-14–knockout mice

The mouse strain with floxed MMP-14 has been described previously (Zigrino et al., 2012). Mice were crossed to a mouse line that expresses the estrogen receptor inducible Cre recombinase under the control of a fibroblast-specific regulatory fragment of the pro-α2 (I) collagen gene (Sonnylal et al., 2007, Zheng et al., 2002). Cre activity was induced in all animals including controls starting from 4 weeks of age by feeding with tamoxifen (400 mg/mg pellets; Haarlan, Venray, The Netherlands) for 4–5 weeks. Animals were housed under specific pathogen-free conditions in a 12-/12-hour light/dark cycle with free access to food and fresh water ad libitum. Mice were killed by carbon dioxide. For experimental skin fibrosis we used an established mouse protocol (Yamamoto et al., 1999); further information is provided in the Supplementary Materials online. All animal experiments were performed in compliance with German Regulations for Welfare of Laboratory Animals and were approved by the Regierungspräsidium Köln, Germany (NRW authorization 50.203.2-K 37a, 20/05 and AZ2010.A342).

All additional experimental procedures are described in detail in the Supplementary Materials and Methods.

### Tensile strength measurements

Full-thickness (hourglass-shaped form, width = 5 mm in middle and 10 mm at the ends, length = 25 mm) stripes of skin from 13-week-old tamoxifen-induced mice (T2) were used to determine the tensile strength by a material testing machine (Z2.5/TN1S; Zwick GmbH & CoKG, Ulm, Germany) with a load cell of 100 N (Lettmann et al., 2014). Samples were fixed between two rippled clamps. After preloading (0.05 N, 0.1 mm/s), skin was stretched with a crosshead speed of 15 mm/min until failure. To calculate the stress, skin thickness was assessed in sections prepared for histological analysis. Stiffness and Young’s modulus were determined from the slope of the linear portion of the load-deformation and stress-strain curves, respectively. Detailed information on the parameters used is provided in the Supplementary Materials.

### Processing of tissues

The acetic acid extraction of collagen was performed as previously described (Asano and Trojanowska, 2013). Skin punches (6 mm) were taken from the back of each mouse (sex and age matched), minced, and incubated in phosphate-buffered saline overnight at 4°C with stirring. Tissue was harvested by centrifugation at 12,000*g* for 15 minutes and suspended in 10 volumes of cold 0.5 mol/L acetic acid with the addition of pepsin (1:10 ratio of pepsin to tissue wet weight). Extraction was performed overnight at 4°C with stirring, and supernatant was dialyzed against 0.1 mol/L acetic acid. Specimens were stored at –20°C before processing.

### Cell collagen degradation assay

The capacity of cells to degrade type I collagen fibrils was assessed as described (Holmbeck et al., 1999). Twenty-four multiwell plates were coated with a film of reconstituted rat tail tendon type I collagen fibrils, and fibroblasts from MMP-14^Sf–/–^ and control mice were cultured on top of this layer for 5 hours. After washing 3 times in phosphate buffered saline, cells were incubated for 5 days in serum free Opti-MEM I (GIBCO-BRL, Gaithersburg, MD) with or without addition of 10^−9^ mol/L of IL-1β, 10^−8^ mol/L of tumor necrosis factor-α, and 20 μmol/L of GM6001. After incubation, cells were removed with trypsin/EDTA, and the residual collagen fibril film was stained with Coomassie blue (Sigma). Experiments were repeated twice, each using two independently isolated MMP-14^Sf+/+^ and MMP-14^Sf–/–^ fibroblasts (four independent fibroblast lines).

### Statistics

Statistical analysis was performed using GraphPad Prism (GraphPad, San Diego, CA). Two-tailed Student *t* test was used for data analysis, with *P* < 0.05 considered to be statistically significant. For multiple comparisons we used analysis of variance tests.

## Conflict of Interest

The authors state no conflict of interest.
