# Supplementary material for: Fibroblast-Derived MMP-14 Regulates Collagen Homeostasis in Adult Skin
Source: J Invest Dermatol. 2016 Aug;136(8):1575–83. doi: 10.1016/j.jid.2016.03.036 (PMC4946792; doi:10.1016/j.jid.2016.03.036)
Supplement: Supplementary Data [file mmc1.pdf]

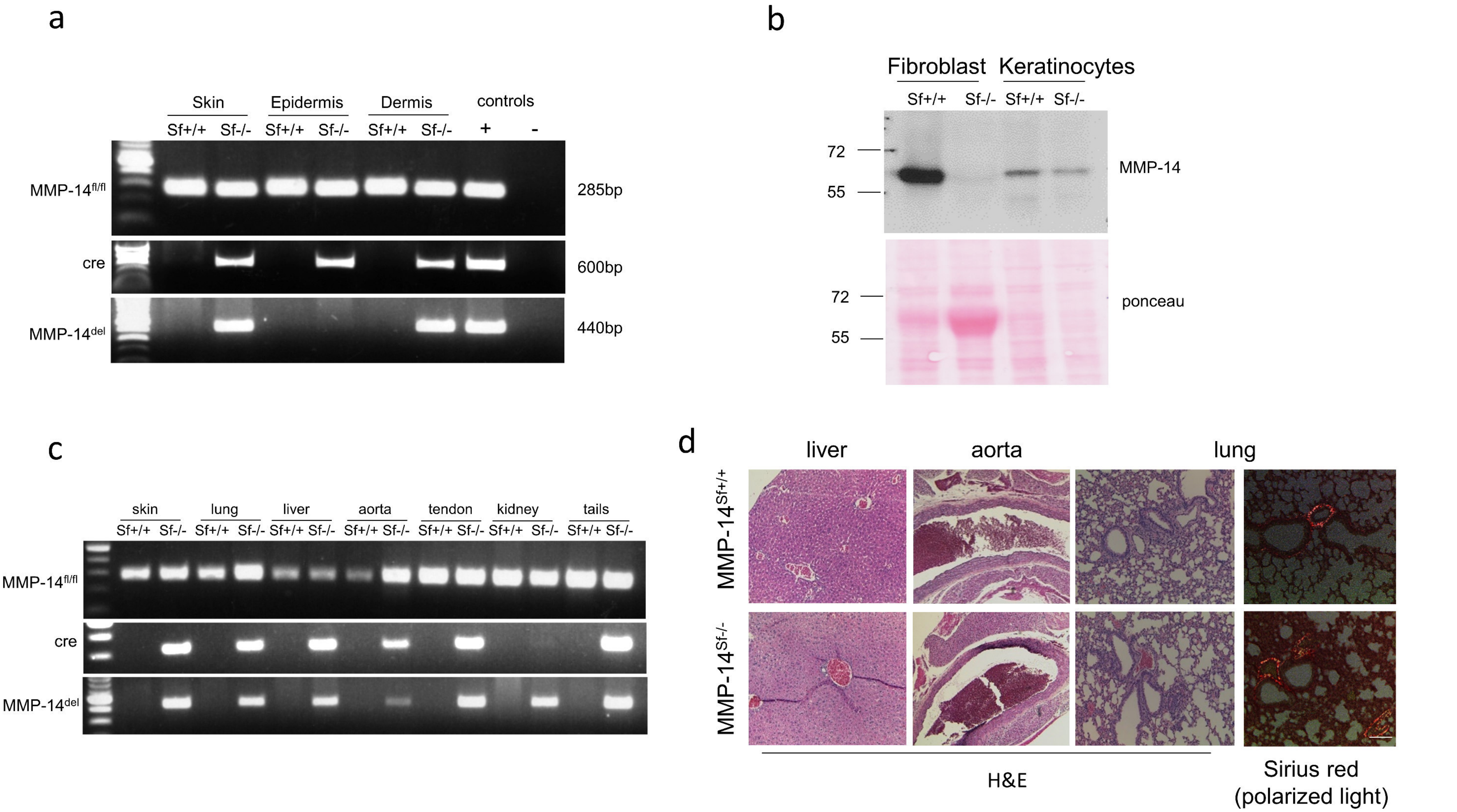

Figure S1

a) Genotyping of epidermis and dermis split skin and **b)** immunoblot analysis of MMP-14 in lysates from fibroblasts and keratinocytes isolated from mouse skin. Ponceau staining of the membrane before immunodecoration shows protein loading and efficient transfer. **c)** genotyping of isolated organs at T2 of MMP-14$^{Sf+/+}$ and MMP-14$^{Sf-/-}$ mice, and **d)** tissue analysis, H&E was used to assess their overall morphology and by Sirius red/analysis with polarized light was used to investigate whether excessive collagen deposition is also detected in these organs.

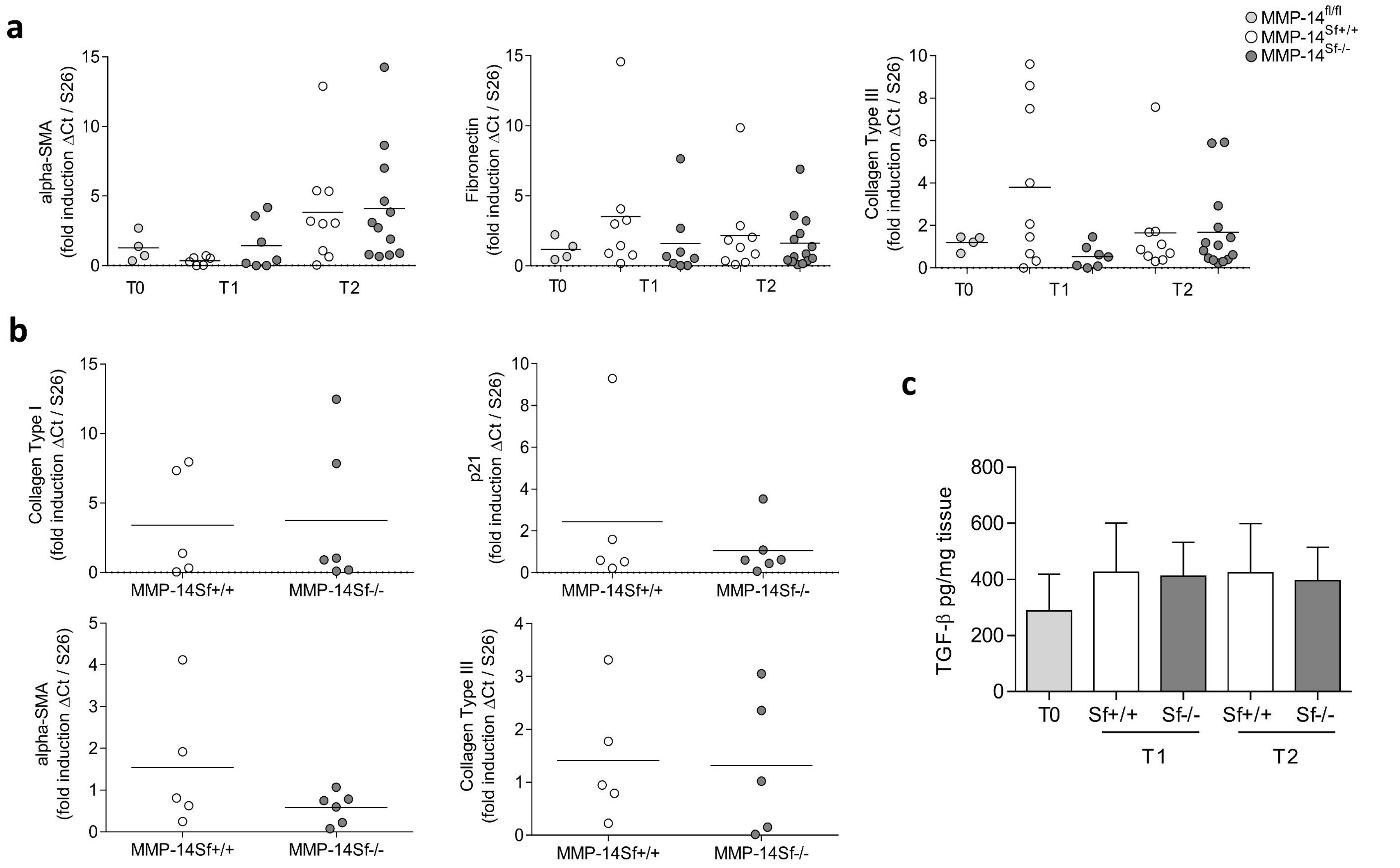

Figure S2

a) Real-time RT-PCR analysis of collagen type I, III and FN transcripts was performed on RNA isolated from skin specimens collected at T1 and T2; **b)** Real-time amplification of collagen type I, III, alpha-SMA and p21 in mouse of 10 months of age. Each dot represents one specimen/mouse. Amplification of S26 was used as a loading control for normalization of amplified transcripts. **c)** TGF-b quantification by Elisa in skin extract (n=3)

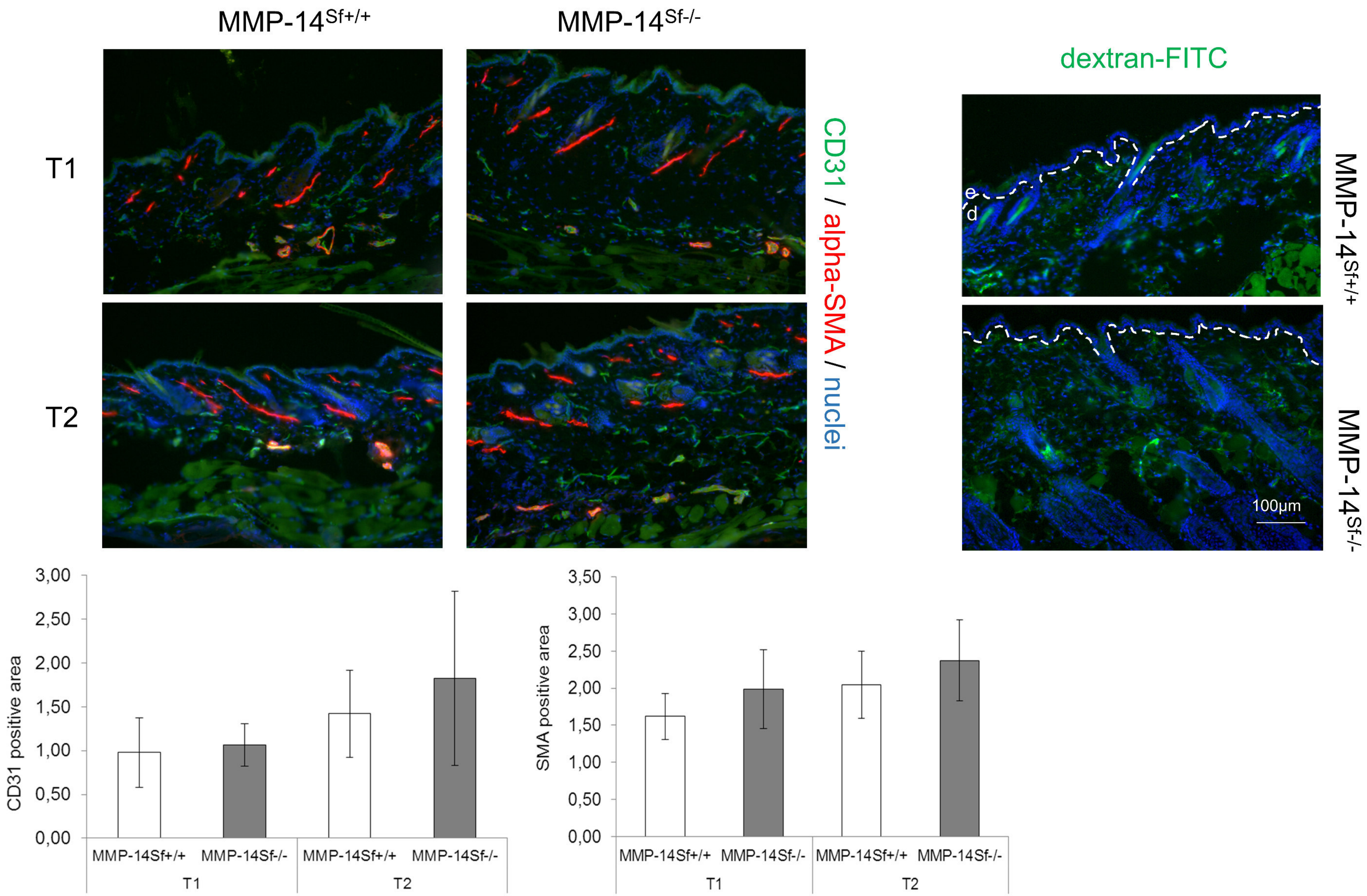

Figure S3

Skin specimens collected at T1 and T2 were stained by immunofluorescence with anti-CD31 and anti-alpha-SMA antibodies to analyze vascular structures and smooth muscle cells in the dermis. Staining was quantified and relative average values shown below (n=4). Nuclei are stained in blue by DAPI. In mice at T2, 2 hours prior collection, dextran-FITC (2000Da) was injected i.v. to analyze vascular permeability in steady state.

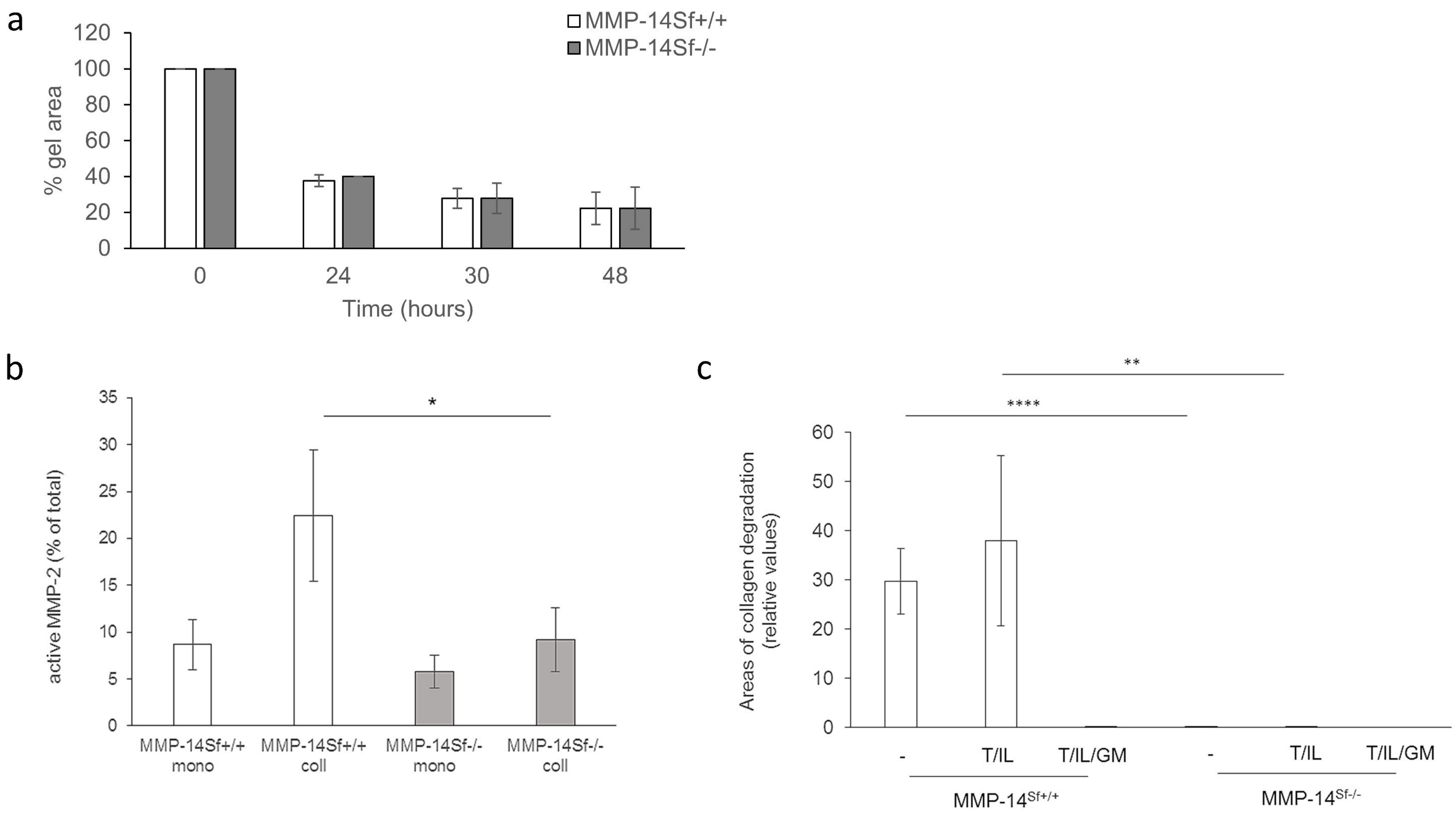

Figure S4

a) fibroblast were embedded in three-dimensional collagen lattices and gel contraction monitored over time. Shown are the average gel sizes as function of initial area set as 100% (average of two experiments performed in triplicates). **b**) gelatinolytic areas of active MMP-2 detected by gelatin zymography (Figure 6) were quantified by imageJ and here expressed as function of the total amount, average ± SD. (n=3) (*p≤0,04;'mono' cells grown as monolayer or 'coll' in collagen lattices) **c**) collagenolytic activity (white areas; Figure 6) of fibroblasts seeded on a reconstituted type I collagen film in the absence (-) or presence of TNF, IL1 (T/IL) or GM6001 inhibitor (T/IL/GM) was quantified by image analysis and displayed here as average ± SD. (****p≤0,0001;**p≤0,001).

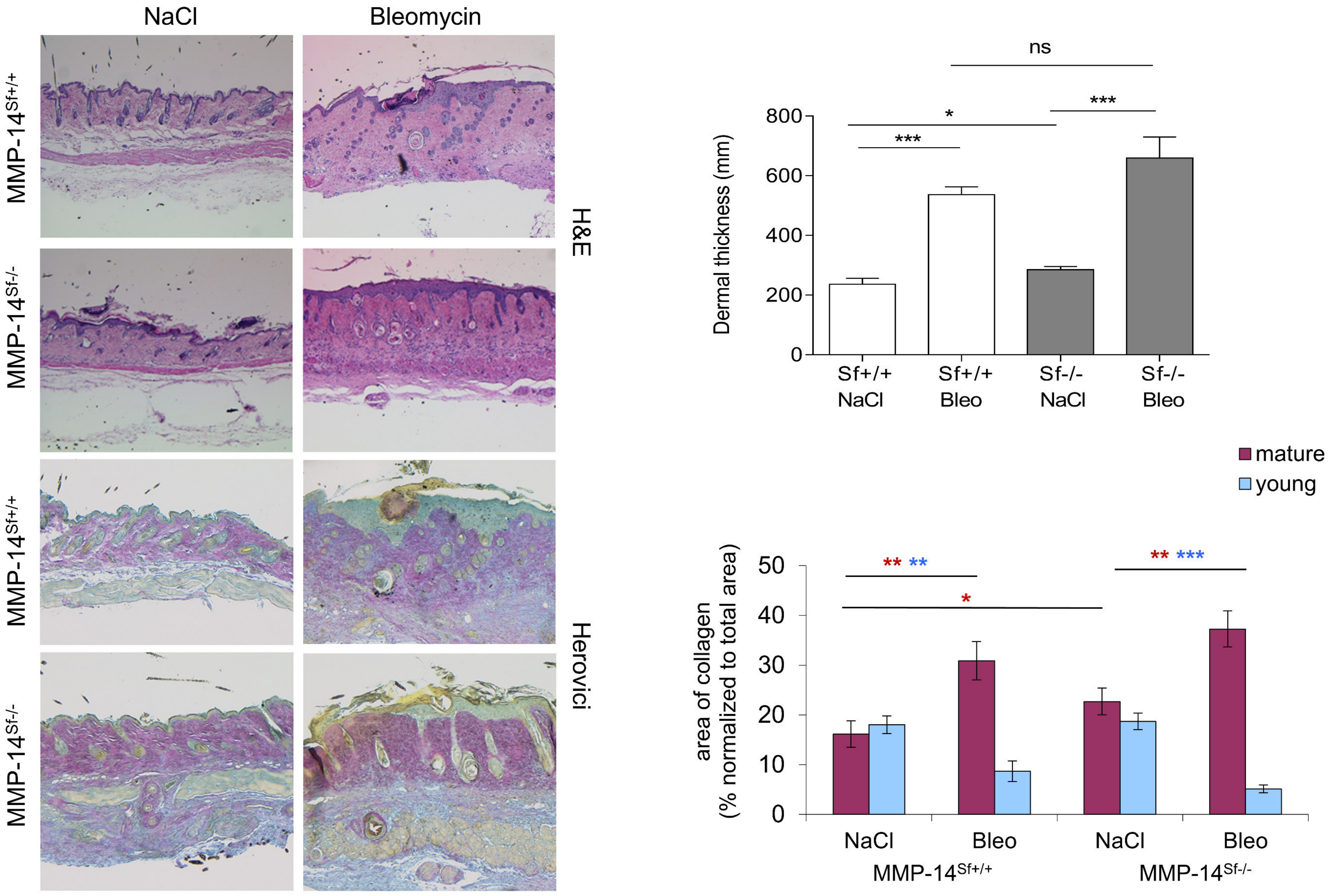

Figure S5

Analysis of dermal thickness and quantification of Herovici staining was performed on control-treated (NaCl) and bleomycin-induced fibrotic lesions. Average dermal thickness in MMP-$14^{Sf+/+}$ (Sf+/+) and MMP-$14^{Sf-/-}$ (Sf-/-) is shown on the upper right.

Quantification of the average blue (young collagen) and purple (mature collagen) is shown below on the right (n≥7 / genotype); *p≤0,03; **p≤0,008; ***p≤0,001)

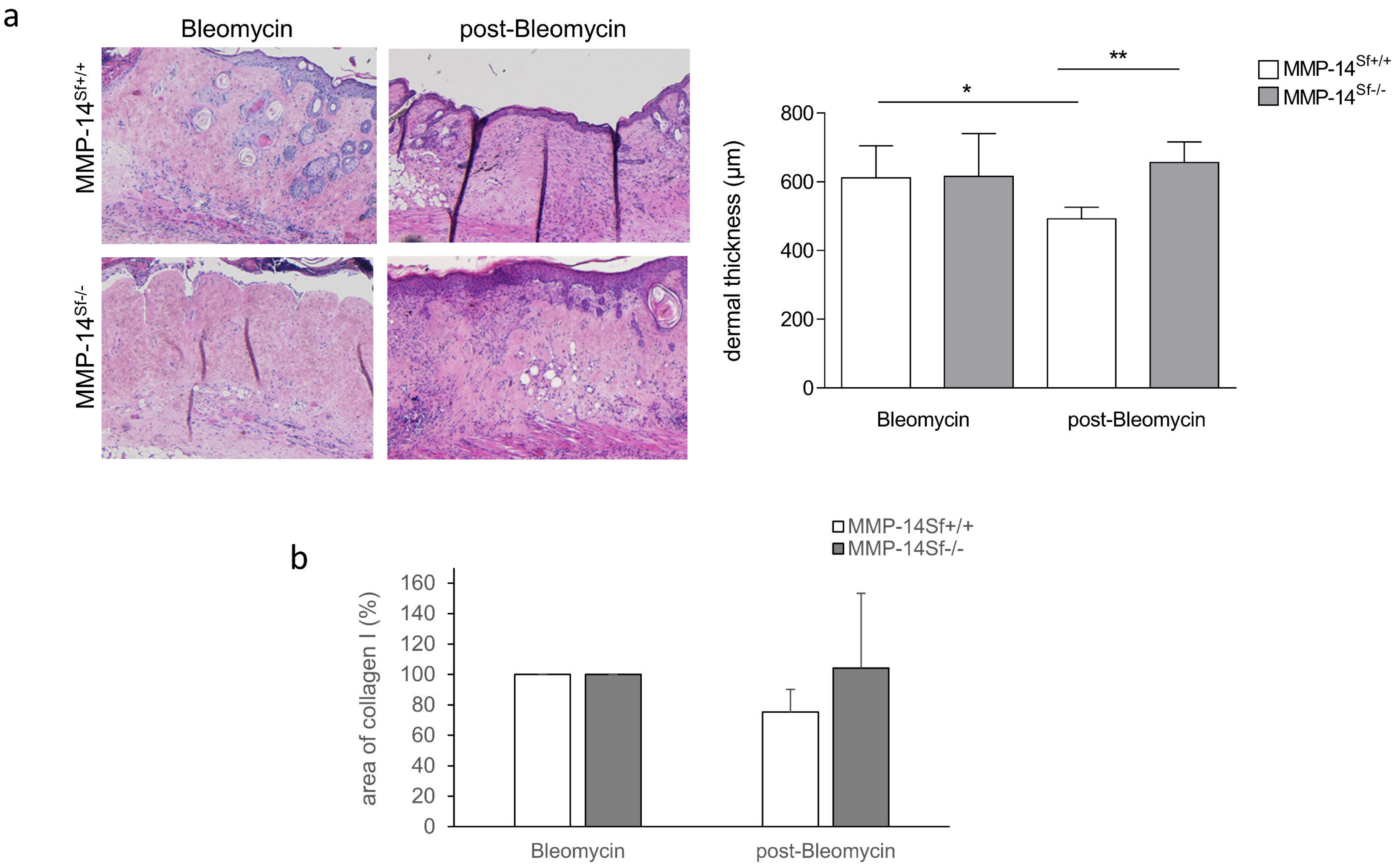

Figure S6

Analysis of dermal thickness (**a**) and quantification of collagen type I by Herovici staining (**b**) were performed on control-treated (NaCl) and bleomycin-induced fibrotic lesions after a resolution time of 4 weeks post Bleomycin treatment. Average dermal thickness in MMP-$14^{Sf+/+}$ (Sf+/+) and MMP-$14^{Sf-/-}$ (Sf-/-) is shown on the left.

Quantification of the average collagen type I is shown on the right (n ≥ 4 / genotype); *p≤0,04; **p≤0,0019)

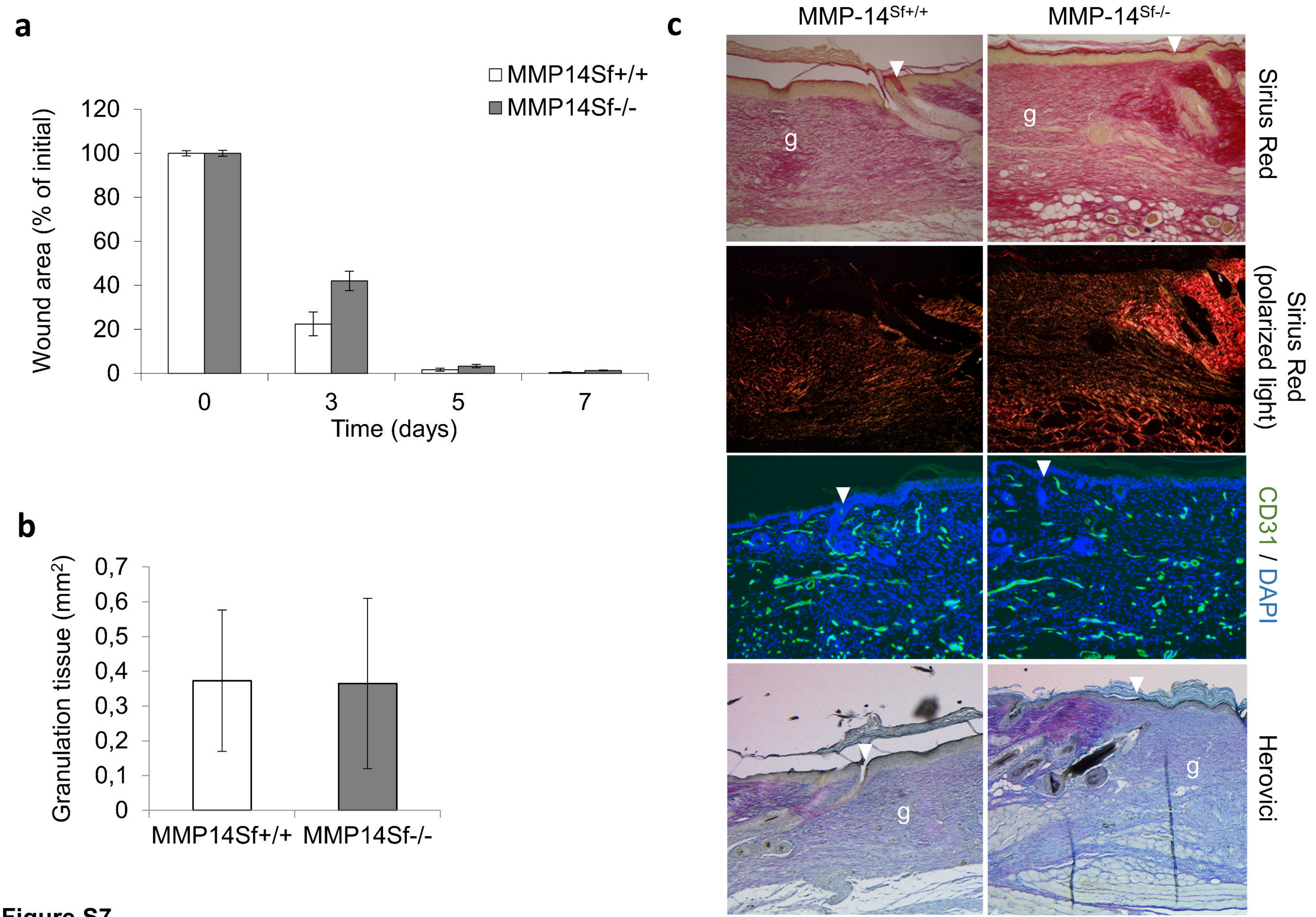

Figure S7

a) Wound closure was quantified over time and the macroscopic open area was measured after wounding and calculated as percent of the initial surface area (average ±SD) (n=4). **b)** Amount of granulation tissue (g) formed at day 13 was quantified on sections. **c)** Excised wound at day 13 were stained with sirius red and by Herovici to qualitatively asses newly deposited collagen. Newly formed and invaded vascular structures in the granulation tissue were detected using an anti-CD31 antibody. Nuclei are in blue. White arrowhead mark the original wound border.

Table SI

Gene		Primer-seq. (5' - 3')	amplified fragment
MMP-8	for	GGCAGAGGAAGGCAGGAGAGG	484bp
	rev	AAGGTCAGGGGCGATGCTACAC	
MMP-13	for	CTGGTCTTCTGGCACACGCT	610bp
	rev	GCAGCGCTCAGTCTCTTCAC	
MMP-15	for	GACAGCTCACCGTTTGATGG	538bp
	rev	ACACAAACATCTCTCCGCGG	
MMP-16	for	GGAGACAGTTCCCCATTTGA	196bp
	rev	CGTTGGAATGTTCCAGTCCT	
Coll Type I (alpha1)	for	TGCCGTGACCTCAAGATGTG	442bp
	rev	CACAAGCGTGCTGTAGGTGA	
alpha SMA	for	CAGCGGGCATCCACGAA	218bp
	rev	CCCACCGATCCAGACAGA	
Fibronectin	for	TGATCATGCTGCTGGGAC	197bp
	rev	CTCGGTTGTCCTTCTTGCTC	
Coll Type III	for	GCGGAATTCCTGGACCAAAAGGTGATGCTG	230bp
	rev	GCGGGATCCGAGGACCACGTTCCCCATTATG	

Primer sequences used for real-time amplifications.

Supplementary Material and Methods

Genotyping

Genotyping of animals was performed by PCR on genomic DNA obtained from tail and tissues biopsies as previously described (Zigrino *et al*., 2012; Klose *et al*., 2013). The deletion was confirmed by PCR amplification using primers located outside of the LoxP sequence ("del"). These primers matched 100% only in the designed locations and amplified a gene fragment of 440bp only following Cre-mediated recombination leading to depletion of the "floxed" gene fragment. Primers used were as follows: for the wild type allele and the floxed allele: 5´GAG GCA GAG GCA GAA CAA GC 3´, 5`GAG CAT CAG AAA GTT GAG AGG 3´; Cre recombinase amplification: 5´GAC GGA AAT CCA TCG CTC GAC CAG 3´, 5´ GAC ATG TTC AGG GAT CGC CAG GCG 3´; MMP-14-deleted fragment: 5´GAG GCA GAG GCA GAA CAA GC 3´, 5´ CCA CCA AGA AGA TGT CAT TCC 3´.

RNA isolation, RT-PCR and real-time PCR

Total RNA from cells and tissues (6 mm punch biopsies) was isolated using The Qiagen Fibrous Kit according to the provided instructions. For quantitative real time PCR analysis, reversed transcribed RNA was used for real time PCR using the StepOne Real-Time (Applied Biosystems). Five microliter of sample cDNA was added to the master mix and the amplification was performed in a total volume of 20 µl for 40 cycles. The thermal cycling conditions were set to 50°C for 2 min and 95°C for 10 min, followed by 40 cycles of amplification at 95°C for 15 s and 60°C for 1 min for each cycle. S26 was used as a loading control and for normalization of amplified transcripts Primers used for real time amplifications are listed in Supplementary Table 1.

Cell culture and preparation of cell supernatants

Murine fibroblasts were isolated from skin after enzymatic digestion and cultured as previously described with the only difference that in the current study adult mouse skin

specimens were used (Florin *et al.*, 2006; Mauch *et al.*, 2010). Genotypes were routinely confirmed in cultures as prolonged culture of incompletely depleted fibroblast populations could be overgrown by undepleted cells. For preparation of cell supernatants, mouse fibroblasts were seeded in plates and cultured for 4 days, washed with PBS and cultured for further 48 h in serum free medium. Cell supernatants were collected, cleared by centrifugation 5 min at 2000xg, to remove suspended cells and debris. Cleared media were either used directly for analysis or stored at -20°C until use. Cell monolayer were collected by scraping directly in lysis buffer (RIPA buffer, 150 mM NaCl, 50 mM Tris-HCl, 1% NP-40, 0.1% SDS, 0.5% Na-deoxycholate, 5 mM EDTA, 1% Triton X-100, pH 8, supplemented with protease and phosphatase inhibitor cocktails (Sigma), and homogenized by MixerMill 3 min at 30Hz. Lysates were centrifuged 30 minutes at 4°C at full speed, transferred to new tubes and stored at -20°C until use. Gelatin zymography was performed as previously described (Zigrino *et al.*, 2001).

Protein analysis

Equal amounts of lysates or of supernatants normalized to cell lysates were fractionated by SDS-PAGE under reducing conditions and transferred onto Hybond-C Super™ (Amersham Pharmacia Biotech). After blocking non-specific binding sites with 5% skimmed milk (w/v) in PBS containing 0.5% Tween (v/v), the blots were incubated with the primary antibodies overnight at 4ºC. Bound primary antibodies were detected using a horseradish peroxidase-conjugated secondary antibody (1:2000; Dako, Hamburg, Germany) and visualized with the ECL system (ECL™; Amersham Pharmacia Biotech). For immunodetection of MMP-14, peptide antibodies were described before (Zigrino et al., 2012). Collagen type I antibodies were from Quartett. Actin was detected using a mouse monoclonal antibody (MP Biomedicals, Irvine, CA).

Levels of mouse $TGF\beta_1$ were determined by ELISA of tissue lysates, with or without acid-activation, according to manufacturer's procedures (R&D Systems). Four tissues samples per genotype were analyzed and all samples were analyzed in duplicate.

Cross-links analysis

For cross-link analysis, specimens were reduced by sodium borohydride (Sigma, Germany; 25 mg $NaBH_4$/ml in 0.05 M NaH_2PO_4/0.15 M NaCl pH 7.4, 1 h on ice, 1.5 h at room temperature). Specimens (about 20 mg wet weight) were digested with high purity bacterial collagenase (C0773; Sigma, Germany; 50 U/ml, 37° C, 12 h). After centrifugation, the soluble fractions (collagen cross-links) were hydrolyzed in 6 N HCl at 110°C for 24 h. The hydrolysates were pre-cleared by solid phase extraction to remove the bulk of non-cross linked amino acids (Macherey Nagel, Düren, Germany). Dried eluates were dissolved in sodium citrate loading buffer (pH 2.2) and analyzed on an amino acid analyzer (Biochrom 30, Biochrom, Great Britain) using a three buffer gradient system and post column ninhydrin derivatization. The column was eluted for 5 min (flow rate 15 ml/h) with sodium citrate buffer (pH 4.25), for 40 min with sodium citrate buffer (pH 5.35) and for 20 min with sodium citrate/borate buffer (pH 8.6) at 80° C. Retention times of individual cross-links were established with authentic cross-link compounds. Quantitation was based on ninhydrin generated leucine equivalence factors (DHLNL, HLNL: 1.8; HP, LP: 1.7). The nomenclature used in this paper refers to the reduced variants of difunctional intermediate cross-links (DHLNL, HLNL, HHMD).The collagen and the protein content of the specimens were analyzed in an aliquot of hydrolyzed specimens of the soluble and the residual fractions prior to solid phase preclearance. Collagen content was calculated based on a content of 14 mg hydroxyproline in 100 mg collagen.

Histological and indirect immunofluorescence analysis of skin sections and cells

Cryosections (6 µm) of the dorsal skin were stained with hematoxylin and eosin, and documented using a Leica (DMLB) microscope. Cryosections of normal dorsal skin were fixed for 2 min (CD31, αSMA; from Dako and Abcam, respectively) in cold acetone. Immunofluorescence detection was performed as previously described (Mauch *et al.*, 2010; Zigrino *et al.*, 2012). Herovici's staining was performed on paraffin embedded tissue sections as previously described (Collins *et al.*, 2011). Blue and red colours from Herovici staining on recordered images were inverted and split into single channels to separately assess the amount of blue (immature) or purple (mature) collagen fibrils. Quantification was performed using ImageJ software (http://rsb.info.nih.gov/ij). Cells cultured on glass coverslips were fixed in cold aceton, dried and after rehydration, and blocking were incubated with primary antibodies overnight (collagen type I and PDGFRα, from Abcam and R&D systems, respectively). Electron microscopy and analysis of fibril diameter was performed as described elsewhere (Starborg *et al.*, 2013; Taylor *et al.*, 2015).

Tensile strength measurements

Stiffness and the Young´s-modulus measured the resistance of the material to elastic deformation under load. The stiffness ($\kappa = F/d$) is defined as the ratio of force divided by the deformation and was determined from the slope of the linear (elastic) part of the force-deformation curve. The Young´s-modulus (elastic modulus; $E = \sigma/\varepsilon$) is defined as the ratio of stress (force/area) divided by the strain (deformation/initial length) and was determined from the slope of the linear (elastic) part of the stress-strain curve. Thus, stiffness is a structural property and the Young´s-modulus is a material property independent from the geometry of the analyzed material. Further, strain is defined as deformation related to initial length (determined at pre-load).

Experimental skin fibrosis and tissue repair experiments

For experimental skin fibrosis we used an established mouse protocol (Yamamoto *et al.*, 1999). Eight to nine weeks old tamoxifen-induced mice (sex and age matched) received repeated intradermal injection of bleomycin sulphate (100 µg of bleomycin in 0.9% NaCl; Medac) into the back skin after anaesthetization and shaving on 5 days/week for 4 weeks. Tissues were fixed in 4% formalin or embedded in O.C.T.™ Compound (Tissue Tek, Vogel, Giessen, Germany).

Wound healing experiments were performed as previously described (Zigrino *et al.*, 2012). Briefly, 8-9 week old tamoxifen-induced mice (sex and age matched) were anesthetized, and after shaving, two full-thickness excisional wounds (4 mm diameter) were generated on the back of each animal by excising skin and the *panniculus carnosus*. Animals were sacrificed when wounds were completely repaired and a small scar visible. Wounds were bisected in a caudo-cranial direction and embedded in OCT compound or parraffin.

References not included in the main text

Starborg T, Kalson NS, Lu Y*, et al.* (2013) Using transmission electron microscopy and 3View to determine collagen fibril size and three-dimensional organization. *Nature protocols* 8:1433-48.

Taylor SH, Yeung CY, Kalson NS*, et al.* (2015) Matrix metalloproteinase 14 is required for fibrous tissue expansion. *eLife* 4.
